# Sea-level rise and archaeological site destruction: An example from the southeastern United States using DINAA (Digital Index of North American Archaeology)

**DOI:** 10.1371/journal.pone.0188142

**Published:** 2017-11-29

**Authors:** David G. Anderson, Thaddeus G. Bissett, Stephen J. Yerka, Joshua J. Wells, Eric C. Kansa, Sarah W. Kansa, Kelsey Noack Myers, R. Carl DeMuth, Devin A. White

**Affiliations:** 1 Department of Anthropology, University of Tennessee, Knoxville, Tennessee, United States of America; 2 Department of Anthropology, Northern Kentucky University, Highland Heights, Kentucky, United States of America; 3 Department of Anthropology, Indiana University South Bend, South Bend, Indiana, United States of America; 4 The Alexandria Archive Institute/Open Context, San Francisco, California, United States of America; 5 Department of Anthropology, Indiana University, Bloomington, Indiana, United States of America; 6 Geographic Information Science and Technology Group, Oak Ridge National Laboratory, Oak Ridge, Tennessee, United States of America; University at Buffalo - The State University of New York, UNITED STATES

## Abstract

The impact of changing climate on terrestrial and underwater archaeological sites, historic buildings, and cultural landscapes can be examined through quantitatively-based analyses encompassing large data samples and broad geographic and temporal scales. The Digital Index of North American Archaeology (DINAA) is a multi-institutional collaboration that allows researchers online access to linked heritage data from multiple sources and data sets. The effects of sea-level rise and concomitant human population relocation is examined using a sample from nine states encompassing much of the Gulf and Atlantic coasts of the southeastern United States. A 1 m rise in sea-level will result in the loss of over >13,000 recorded historic and prehistoric archaeological sites, as well as over 1000 locations currently eligible for inclusion on the National Register of Historic Places (NRHP), encompassing archaeological sites, standing structures, and other cultural properties. These numbers increase substantially with each additional 1 m rise in sea level, with >32,000 archaeological sites and >2400 NRHP properties lost should a 5 m rise occur. Many more unrecorded archaeological and historic sites will also be lost as large areas of the landscape are flooded. The displacement of millions of people due to rising seas will cause additional impacts where these populations resettle. Sea level rise will thus result in the loss of much of the record of human habitation of the coastal margin in the Southeast within the next one to two centuries, and the numbers indicate the magnitude of the impact on the archaeological record globally. Construction of large linked data sets is essential to developing procedures for sampling, triage, and mitigation of these impacts.

## Introduction

In recent years, concerns about the damaging effects of anthropogenic global climate change have been amplified by the increasing frequency of destructive weather events, large-scale wildfires and droughts, and a growing body of evidence indicating sea levels will rise appreciably over the next several centuries, from 1 m in the next century to 5 m or more in the centuries thereafter ([1–4]). The effects of such increases in sea level will be severe and long-lasting. At present, over 40% of all people worldwide live within a 100 km distance from the nearest coastline, many in low lying areas vulnerable to sea level rise [5–11]. Should projected rises occur, the effect on humans living on and near the coast, including the loss of infrastructure is nearly incalculable, and will require population movement and resettlement on scales unprecedented in human history. Here we demonstrate, using examples from the southeastern United States, that not only modern populations and properties, but also irreplaceable heritage in the form of the physical record of past human settlements, are currently vulnerable to projected sea level rise as a destructive agent. We argue that archaeologists and society at large should direct increased attention to planning for and mitigating these losses to heritage resources.

The worldwide historic preservation community has begun to express serious concerns over the threat of global climate change to the archaeological and historic record, especially with respect to the potential loss of data that will occur as coastal zones are subjected to increased erosional forces and inundation from rising sea levels [12–35]). Analyses have been directed to determining how rising or fluctuating sea levels damage archaeological and historical resources, sacred and traditional sites, as well as submerged resources like former terrestrial archaeological sites, buildings, and shipwrecks [36–45]. Threats to coastal and near-coastal cultural resources will also come from activities undertaken to resist rising waters. Sea walls and other barriers may provide protection to critical coastlines at favorable cost benefit tradeoffs [46], but their construction will potentially impact large numbers of existing and undocumented cultural resources, far exceeding work conducted as a result of recent oil spills like the Exxon Valdez in Alaska and Deepwater Horizon along the southeastern Gulf coast [47–50]. Far less consideration has been given to the damage or loss of cultural resources that will occur as populations residing in coastal areas are displaced inland, building new communities or expanding existing ones. In previously less-developed regions, where little prior archaeological work has occurred, innumerable unrecorded archaeological and historical sites will also be threatened. The salvaging of valuable materials from threatened infrastructure itself will likely take a toll on historic properties, although some of the more iconic buildings may themselves be relocated to higher ground. For example, the White House or the Lincoln Memorial may be moved from Washington, D.C., much like the Egyptian New Kingdom era temple of Abu Simbel was moved before the rising waters of the Aswan High Dam submerged the area in the 1960s, and the Cape Hatteras lighthouse was relocated 2,900 feet to protect it from encroaching seas in 1999 [32, 51, 52].

Damage from shoreline erosion represents a significant concern to preservationists, with appreciable research globally now being directed to well-known archaeological and historical resources threatened by such processes [19, 24, 25, 27–45]. Important research is addressing the threat of global climate change, particularly sea level rise, to national landmarks or national parks in the United States [19, 21, 28, 29, 32, 53, 54]. However, more inclusive and geographically broad-based analyses are rare, because comprehensive data sets encompassing all known archaeological and historical resources at regional or continental scales have not previously been available. In the United States, cultural resource data are managed at the state rather than the national level, or within specific federal agencies, making such database development and large-scale analyses challenging. Integrating these data together is crucial to determining how climate change, including fluctuations in sea-level, will impact heritage resources at regional and continental scales. Calls for such syntheses have recently appeared [55, 56], and fostering research on this scale is widely heralded as a grand challenge facing the archaeological profession in the United States [57, 58], essential to exploring questions about changes over time in organizational complexity, human responses to climate change, and long-term settlement dynamics [31, 59–70].

## Linked database development: The DINAA project

**Fig 1:**
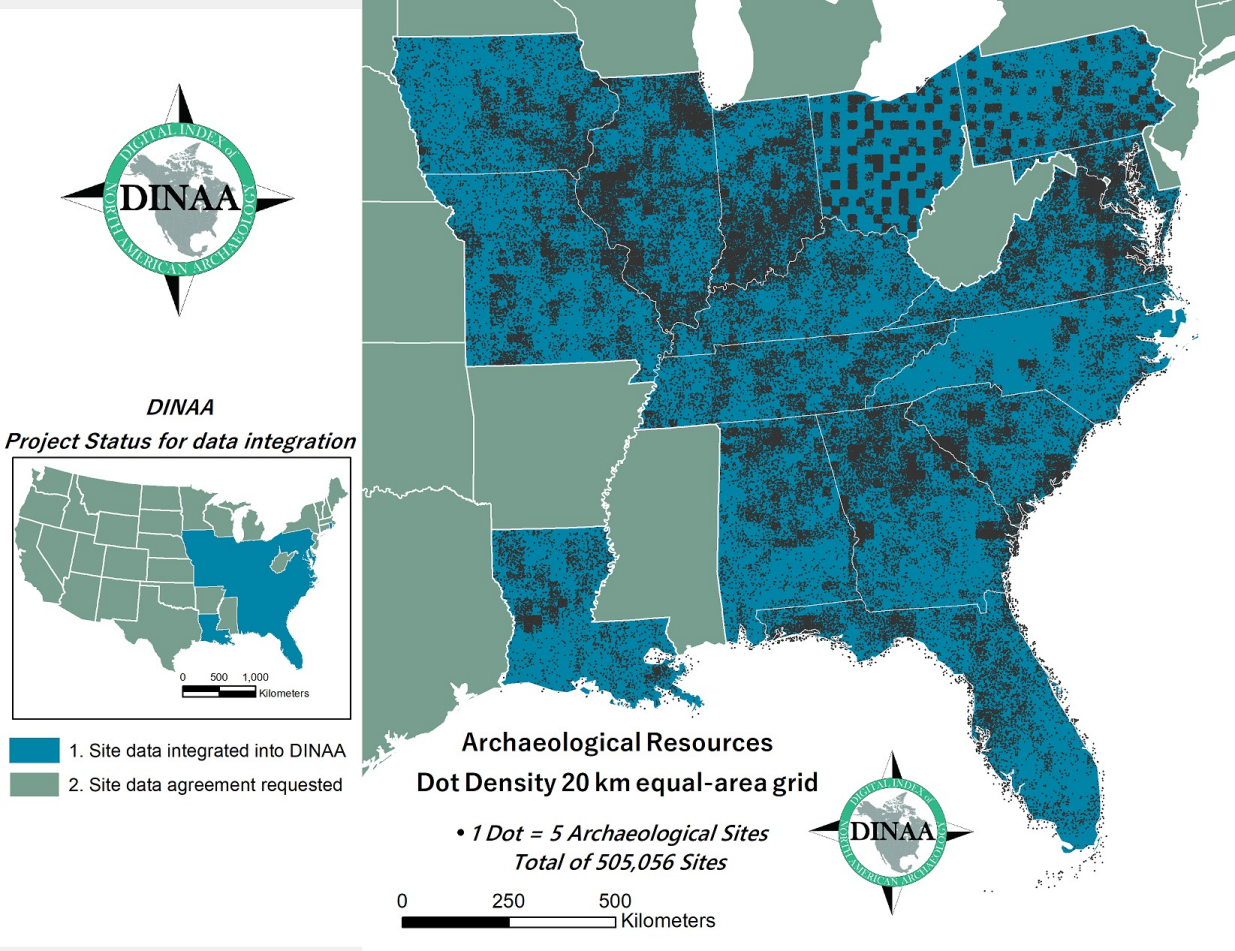
DINAA partnerships as of July 2017 with dot density plot showing distribution of cultural resources at low resolution within states whose data have been received thus far. **Data: [73].** Ohio and most Pennsylvania site data is at county-level resolution.

The Digital Index of North American Archaeology [71–73], or DINAA, permits the examination of relationships between environmental and cultural resources over large areas, by rendering diverse heritage data sets interoperable, and linking them with natural systems data sets encompassing physiography, biota, and climate in the past, present, and projected into the future. A multi-institutional collaboration, DINAA consists of an online, integrated open-source database of archaeological and other kinds of evidence for North America’s human settlement. Since 2012, DINAA has compiled and rendered interoperable archaeological site file data from 15 states in Eastern North America (N = 505,056 sites). This work has been done in consultation and cooperation with government, academic, and tribal stakeholders, and with funding from the National Science Foundation, the Institute of Museum and Library Services, and support from the leadership of archaeological professional organizations, including the Society for American Archaeology, the Society for Historical Archaeology, and the Archaeology Division of the American Anthropological Association [73] (Fig 1). As of October 2017, personnel from 21 states are actively participating in DINAA development, and the project has initiated discussions with site file managers and governing authorities in the remaining 28 states in continental North America, and in other countries, with the goal of developing a truly continental database. Information rendered accessible through DINAA is seeing increasing attention and use by researchers and resource managers, enhancing public awareness, education, and appreciation for scientific research in general and archaeology in particular [74–86].

DINAA is a publicly accessible compilation of existing archaeological site file, collection, and report data from multiple regional, state, and local repositories, linked with other archaeological databases as well as modern and paleoenvironmental data sets, with site numbers serving as the basic identifier and standardized temporal metadata as a relational control between data sets, to permit analyses by selected time periods. Archaeological site files contain data and metadata about the chronology, location, and function of sites, in combination with other information that can include diagnostic artifact descriptions, radiocarbon and other absolute dating determinations, and bibliographic citations. While each state and agency uses somewhat different systems, they are rendered interoperable through DINAA.

Through deployment on Open Context [73], an open data publishing service for archaeology, DINAA embraces current best practices in scientific data-management including open standards and open licensing, transparent version control of both data and source code, Linked Data, and iterative development. Through aggregation and human editorial processes to align data set schemas and controlled vocabularies, DINAA provides some of the benefits of centralization without requiring different (and typically financially constrained) state agencies to change their own systems. Thus, DINAA fosters independent development and experimentation through integration of distributed systems managed by a host of institutions. This approach enables community-wide participation and investment in archaeological informatics, making the resulting cyberinfrastructure products shared and useful for all.

DINAA also strictly conforms to legal requirements regarding the maintenance and use of cultural resources data. While analyses like those reported herein can occur making use of records with specific geospatial data, the data itself and permission to use it must be obtained from the agencies maintaining the information. DINAA, accordingly, does not publish or store precise site coordinates online, and the project redacts other sensitive attributes, particularly property ownership, from state site file repositories, in consultation with agency and other interested parties, including tribal nations. Directions to offices to contact to obtain such information for each site are provided with analytical output, but DINAA itself does not maintain or release such data. For public display purposes DINAA site data is aggregated within a tiled web map in Open Context, where a map-tiling algorithm allocates each site record to a 0.176 degree grid cell in the WGS Web Mercator projection (roughly 20x20 km at the equator). The Open Context platform provides publicly accessible online map interfaces for visualization and queries at a low level of spatial resolution that still has great utility when examining distributions encompassing large areas or time periods. DINAA digital data are archived with the California Digital Library, and mirrored in repositories in other countries to ensure long term survival [71, 72].

**Fig 2:**
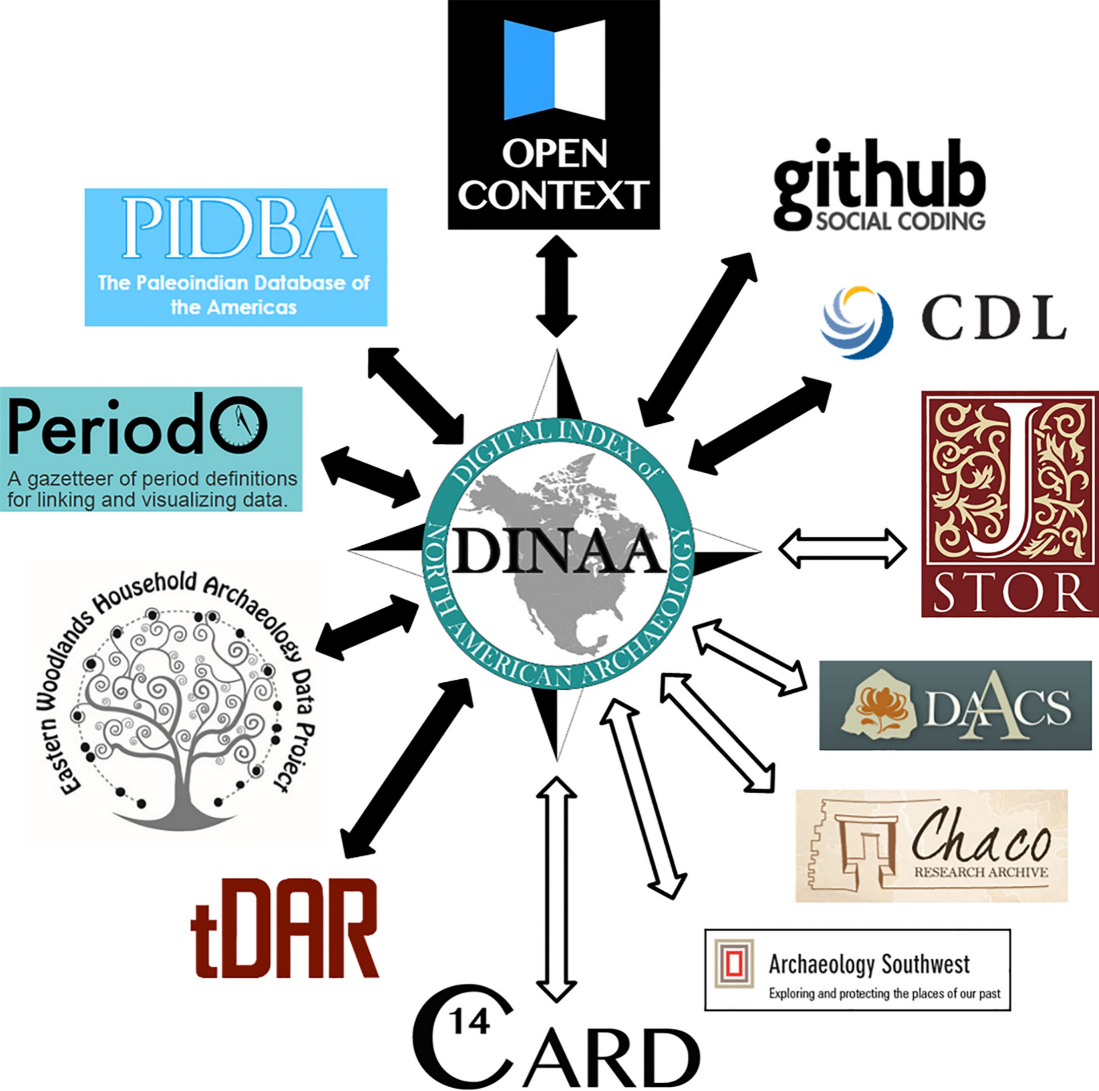
DINAA links information in a wide range of online data repositories, using archaeological site numbers as the common referent. DINAA directs users to these outlets, but access and content control remains on their systems (black arrows indicate existing linkages, white arrows indicate linkages under development).

Indexing, or linking to and rendering interoperable data from many sources and across disciplines is a major function of DINAA, increasing its utility for resource management, research, and public education (Fig 2). By cross-referencing distributed collections on the Web, DINAA enables users to find and access relevant content in archaeological systems like Archaeology Southwest [87], the Paleoindian Database of the Americas [88, 89], the Eastern Woodlands Household Archaeology Database Project [90], the Canadian Archaeological Radiocarbon Database [91], the Digital Archaeological Archive of Comparative Slavery [92], the Chaco Research Archive [93], PeriodO [94], and The Digital Archaeological Record [95]. Aggregators such as Pelagios [96], a collaborator with DINAA, can then “harvest” cross-references between different systems to present users with services, maps, and visualization tools to discover related data and other media that relate to DINAA curated site files. DINAA can serve as a key node in connecting North American archaeological data, allowing, for the first time, its linkage across multiple time periods and geographic regions, and using an array of environmental data sets to explore fundamental issues such as changes in human land use over time; the nature of the archaeological record collected over the past century, including the identification of research strengths and gaps; and, as we show here, how future changes in climate will affect site preservation and heritage management.

## Impacts of sea-level rise in the southeastern United States

**Fig 3:**
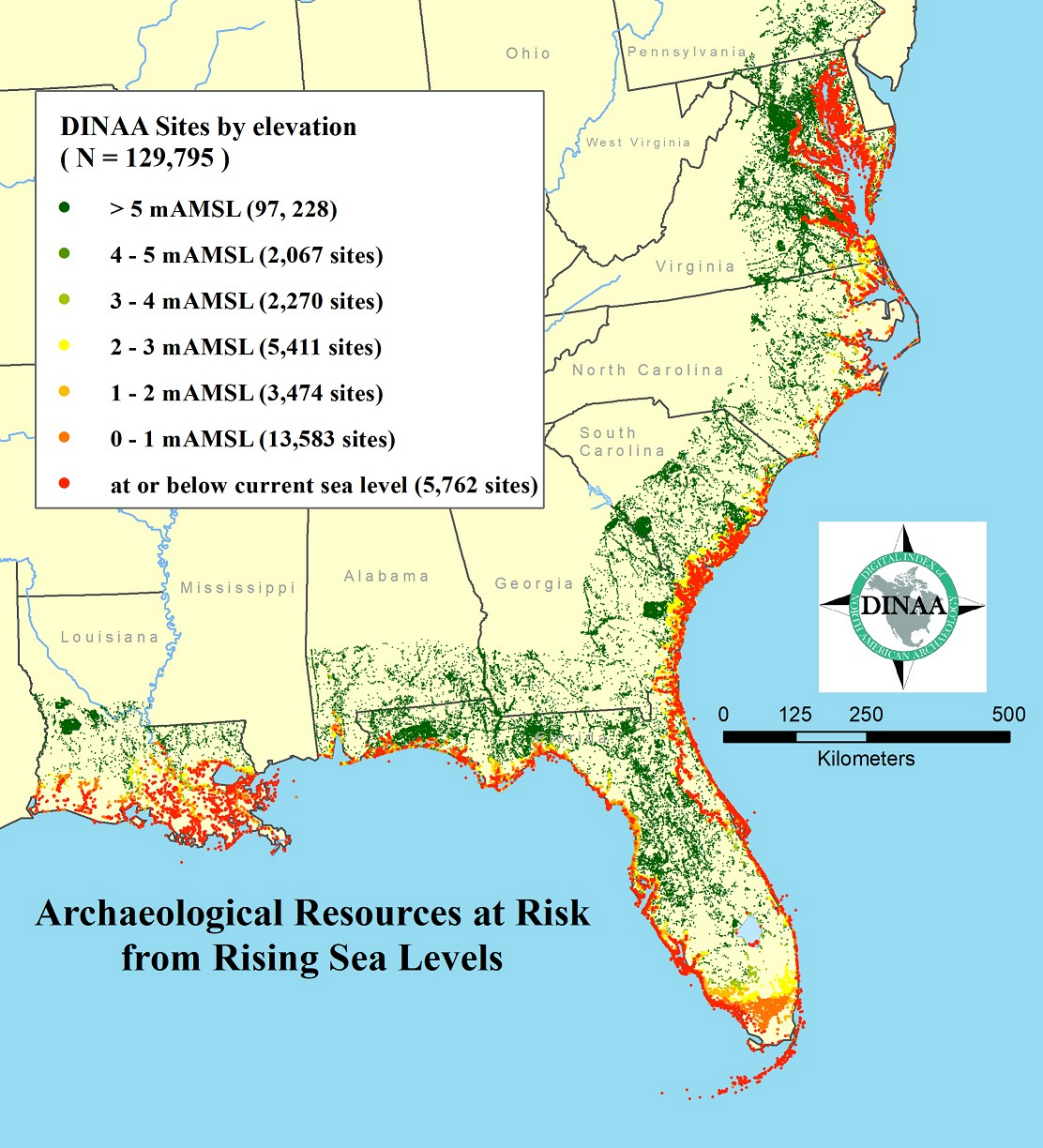
Site incidence as it relates to potential loss from sea-level rise, grouped by elevation in meters above present mean sea level. **Data: [72, 73, 151].** All recorded sites within a buffer of 200 km from the present coastline are shown.

The focus for this study is the southeastern United States, where a vast shoreline characterized by minimal vertical relief exists, and where minor fluctuations in sea level have been shown to have significant effects on shoreline movement and human settlement in the past (e.g., [41, 60–62, 66–70, 97–99]). The southeastern United States is also where DINAA data are most complete. This study draws on archaeological site records from eight states, encompassing most of the recorded archaeological sites on the Atlantic and Gulf coasts of the southeastern United States (n = 129,795 sites; Fig 3). The analysis spans the area from Maryland to the Texas-Louisiana border, and makes use of all recorded sites within these states as of January 2016, including historic properties determined eligible for the National Register of Historic Places (NRHP)[100]. These data were used to develop a GIS-based inventory and assessment of threats to known archaeological and cultural resources located along the Atlantic and Gulf coasts of the eastern United States. Only archaeological site data from Mississippi is not included, due to delays in data transfer. Fortunately Mississippi occupies only a small area along the Gulf coast, and data were available from it for the other analyses conducted.

Three areas of concern raised by projected sea level rise are examined: (1) the numbers of archaeological and eligible historic properties affected, (2) the numbers of people displaced, and (3) the kinds of mitigation strategies necessary based in part on the numbers of sites that will be lost by period. No calendar dates are presented beyond general estimates for when sea levels will reach specific elevations. Such determinations are projections at present, with wide ranges depending on circumstances [1, 3, 4].

### Cultural resource loss due to sea-level rise

Archaeological sites and NRHP Eligible properties in the study area are listed by elevation in Tables 1 and 2, data that serves as a proxy for the numbers that will be lost given sea level rise of varying values. Data are provided in summary form by state and 1-meter increments from 0 to 5 meters, and greater increments beyond that, encompassing all sites and NRHP properties within 200 km of the current coastline. The 200 km buffer was used to assess the numbers of recorded properties at various elevations further inland, where populations may be forced to relocate. More specific analyses can be calculated as needed, for example, around inland areas where population relocation may occur, or along portions of the coast where construction of seawalls might be considered.

**Table 1:** Archaeological site loss in the southeastern United States due to sea level rise within 200 km of the coast. Data: [72, 73, 151].

mAMSL	LA	MS	AL	FL	GA	SC	NC	VA	MD	Total
Below 0	324	n/a	37	1,539	266	547	212	1,077	1,760	**5,762**
0 to 1 m	2,376	n/a	98	3,959	678	1,551	687	2,220	2,014	**13,583**
>1 to 2 m	241	n/a	22	1,322	397	619	270	354	249	**3,474**
>2 to 3 m	356	n/a	125	1,275	1,175	1,350	305	632	193	**5,411**
>3 to 4 m	261	n/a	24	874	106	425	179	262	139	**2,270**
>4 to 5m	150	n/a	13	606	139	471	204	330	154	**2,067**
>5 to 10 m	778	n/a	275	3,360	1,343	3,090	1,128	1,258	707	**11,939**
>10 to 15 m	456	n/a	192	3,457	414	909	879	2,792	1,389	**10,488**
>15 to 20 m	218	n/a	154	2,107	875	537	283	646	330	**5,150**
>20 to 25 m	181	n/a	161	3,090	1,350	656	240	521	165	**6,364**
>25 to 30 m	260	n/a	158	3,158	641	646	470	3,822	590	**9,745**
>30 to 50 m	723	n/a	528	5,523	2,427	1,678	984	1,806	997	**14,666**
>50 m	4,481	n/a	2,177	2,031	4,381	3,681	1,726	16,461	3,938	**38,876**
**Total**	**10,805**	n/a	**3,964**	**32,301**	**14,192**	**16,160**	**7,567**	**32,181**	**12,625**	**129,795**

**Table 2:** National Register of Historic Places eligible property loss in the southeastern United States due to sea level rise within 200 km of the coast. Data: [100].

mAMSL	LA	MS	AL	FL	GA	SC	NC	VA	MD	Total
Below 0	11	2	8	136	12	33	19	27	35	**283**
0 to 1 m	196	34	2	302	18	100	119	124	140	**1,035**
>1 to 2 m	39	10	2	210	7	42	26	27	30	**393**
>2 to 3 m	63	17	58	132	22	54	30	47	17	**440**
>3 to 4 m	83	2	2	56	4	7	10	7	16	**187**
>4 to 5m	47	0	4	33	4	8	8	14	16	**134**
>5 to 10 m	166	34	34	153	27	62	72	58	65	**671**
>10 to 15 m	90	0	5	42	25	9	80	116	125	**492**
>15 to 20 m	76	1	5	32	5	15	41	29	51	**255**
>20 to 25 m	45	0	4	45	5	32	35	41	20	**227**
>25 to 30 m	19	2	6	67	11	29	100	234	69	**537**
>30 to 50 m	39	48	36	210	69	99	132	99	123	**855**
>50 m	78	209	117	87	250	346	422	1,059	560	**3,128**
**TOTAL**	**952**	359	**283**	**1,505**	**459**	**836**	**1,094**	**1,882**	**1,267**	**8,637**

It is clear that small increases in sea level will have great consequences on the coastal archaeological record. A total of 32,898 recorded archaeological sites along the southeastern Atlantic and Gulf coastal margin are within 5 m of modern sea level, including 5,762 recorded at or below sea level and 331 for which no elevation data were available in the state site files or that could be determined given the locational data present (Table 1). Assuming current projections hold, and the sea level rises approximately one meter by the end of the century [1, 2], a total of 19,676 currently recorded archaeological sites will be submerged. Since survey coverage is incomplete, the numbers of actual sites impacted will be much higher. Large numbers of recorded sites are within 1m vertical elevation of modern sea level, and the numbers drop off markedly above 3 m across the region. People in the Southeast appear to have lived in close proximity to the coast in recent millennia, at least in terms of elevation [97–99]. Similar losses are indicated when NRHP eligible property data are examined, with 1,318 at or below 1 m in elevation, and 2,472 within 5 m of modern sea level (Table 2). While some archaeological sites are included in the NRHP data, many historic buildings and landscapes are also present. In addition, traditional cultural properties (TCPs) and resource areas important to Native American groups are often identified through characteristics not recognized by the NRHP, and may not be included in counts of cultural resources in coastal areas. Likewise, not all coastal and offshore areas have been thoroughly surveyed for submerged or partially submerged sites, which will likely be impacted by changes in biotic activity, commercial fishing, boat traffic, and overall access given changes in sea level [36–40]. Again, a substantial drop off in NRHP property numbers is evident immediately away from the coast, and especially above 3 m in elevation, another indicator of the importance of immediate coastal margins in human history [22, 101–103].

The data are sobering: projected sea level rise in the current century, as well as in subsequent centuries, will result in the loss of a substantial portion of the record of both pre-Columbian and historic period human habitation of the coastal margin of the southeastern United States. Tens of thousands of historic and prehistoric archaeological sites, and thousands of properties currently designated eligible for inclusion on the NRHP, which include archaeological sites, standing structures, and other cultural property types, will be submerged and hence lost or damaged, as well as underwater resources that will be effected by changes in ocean acidification, currents, and shipping patterns [22, 23, 103–105]. The impact of changing climate on terrestrial and underwater archaeological sites, historic buildings, and cultural landscapes will be massive. Furthermore, not only are these coastal and near-coastal resources threatened by inundation and erosion, but they will also be threatened by efforts to prevent or delay the loss of coastal land through massive infrastructure projects like sea walls, assuming seas rise slowly enough to permit their construction, or they lie in areas not afforded protection by sea walls. While such activities may slow or even halt the inland advance of coastal waters in some areas [46], they would also likely cause significant damage and destruction to existing heritage resources. Because survey coverage oriented toward finding archaeological sites and historic properties is incomplete in many coastal areas, as are efforts to evaluate these sites in terms of NRHP eligibility, these estimates should be viewed as conservative. Below we assess other impacts of sea level rise likely to impact cultural resources, and discuss the implications of these data in planning for the future.

### Population displacement and land area loss due to sea-level rise

**Fig 4:**
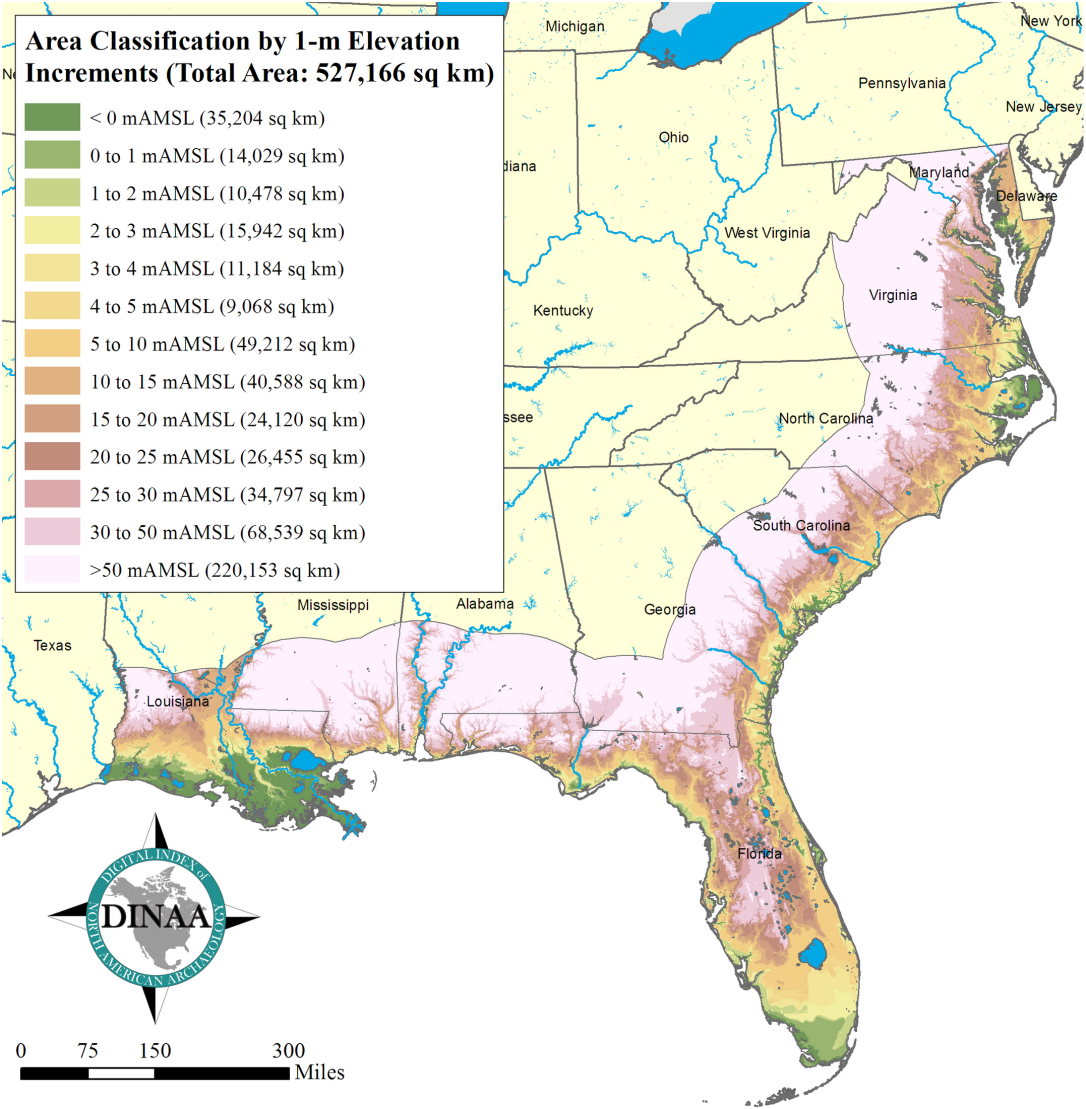
Land area in the southeastern United States within 50 m AMSL. Data: [108].

Sea level rise will displace large numbers of people and inundate large areas on the eastern and Gulf coasts of the United States, even should major construction projects occur to protect critical population and economic centers (Fig 4). Data on the numbers of people and amount of area involved are provided in Tables 3 and 4, encompassing the nine states in the study sample. No areas or population centers are excluded, even those where massive efforts are likely to made to protect them, to provide an accurate determination of the extent of the environmental impact. Population data are derived from 2013 estimates produced as part of the ongoing LandScan initiative undertaken by the Oak Ridge National Laboratory [106, 107]. The 2013 LandScan data set, which is available as a downloadable raster data set, has a horizontal resolution of 1 km2 per pixel. The raster was converted to a point feature, with each pixel from the original raster (and the associated population value per pixel / square kilometer) represented by a single point feature. By spatially joining these points to polygon features representing land areas grouped by elevation into 1-meter increments from 0 to 5 m AMSL (above mean sea level)—derived from 1-arc second (30 m2) resolution digital elevation models provided by the United States Geological Survey’s National Elevation Data set [108]—it is possible to make quantitative predictions about the potential effects of sea level rise on coastal populations. It should be noted that studies excluding tidelands produce different and typically much lower numbers for land area loss [109, 110].

**Table 3:** Population displacement in the southeastern United States due to sea level rise. Data: [106, 107].

mAMSL	LA	MS	AL	FL	GA	SC	NC	VA	MD	Total
Below 0	795,402	10,776	2,363	631,395	18,048	65,574	78,958	377,711	236,880	**2,217,107**
0 to 1 m	273,596	3,381	5,158	634,594	14,434	54,754	49,196	38,663	28,343	**1,102,119**
>1 to 2 m	156,277	2,209	6,827	3,103,454	15,401	68,631	70,352	110,851	34,889	**3,568,891**
>2 to 3 m	236,037	93,461	54,897	2,590,623	138,439	190,086	75,459	501,915	25,428	**3,906,345**
>3 to 4 m	290,289	2,804	4,799	860,511	18,469	52,678	43,948	118,819	14,056	**1,406,373**
>4 to 5m	122,284	4,816	3,148	988,216	39,366	51,792	55,956	102,134	36,307	**1,404,019**
>5 to 10 m	741,792	159,564	87,382	3,258,818	207,995	470,917	495,872	269,773	201,051	**5,893,164**
>10 to 15 m	388,072	49,132	57,471	1,119,765	41,183	157,804	229,663	272,363	415,774	**2,731,227**
>15 to 20 m	224,409	30,278	29,526	805,974	32,840	72,801	160,878	59,859	149,118	**1,565,683**
>20 to 25 m	147,190	9,733	25,433	1,422,249	69,025	123,574	71,594	55,640	81,983	**2,006,421**
>25 to 30 m	57,318	24,005	39,616	1,159,401	49,996	97,076	149,938	477,979	434,498	**2,489,827**
>30 to 50 m	124,471	107,455	194,767	1,837,261	219,256	302,645	553,648	314,064	688,838	**4,342,405**
>50 m	140,078	645,887	673,750	406,486	1,299,360	1,270,284	2,576,182	3,982,484	3,453,346	**14,447,857**
**Total**	**3,697,215**	1,143,501	**1,185,137**	**18,818,747**	**2,163,812**	**2,978,616**	**4,611,644**	**6,682,255**	**5,800,511**	**47,081,438**

**Table 4:** Land area loss in the southeastern United States due to sea level rise, in sq km. Data: [108].

mAMSL	LA	MS	AL	FL	GA	SC	NC	VA	MD	Total
Below 0	18,909	91	73	4,892	1,279	2,194	3,491	1,932	2,342	**35,204**
0 to 1 m	4,334	46	88	6,662	305	312	1,722	268	292	**14,029**
>1 to 2 m	1,619	42	57	5,740	488	360	1,388	400	384	**10,478**
>2 to 3 m	2,399	698	273	6,214	1,326	1,949	2,070	721	291	**15,942**
>3 to 4 m	2,234	45	42	6,793	239	312	985	292	243	**11,184**
>4 to 5m	1,339	41	71	4,580	384	627	1,342	356	327	**9,068**
>5 to 10 m	7,180	1,128	938	22,481	2,529	5,312	6,272	1,666	1,704	**49,212**
>10 to 15 m	7,674	1,279	1,002	11,903	1,366	3,368	8,082	2,879	3,035	**40,588**
>15 to 20 m	3,311	847	995	9,870	1,617	2,388	3,552	1,042	498	**24,120**
>20 to 25 m	2,251	542	958	13,297	1,970	3,802	2,382	1,039	214	**26,455**
>25 to 30 m	2,510	1,273	1,404	11,484	2,188	3,596	4,803	6,324	1,213	**34,797**
>30 to 50 m	5,191	4,688	6,732	19,983	13,795	7,371	7,740	1,703	1,335	**68,539**
>50 m	8,941	32,093	35,259	7,325	42,113	19,047	21,539	43,933	9,905	**220,153**
**Total**	**67,892**	42,814	**47,892**	**131,223**	**69,601**	**50,637**	**65,368**	**62,557**	**21,784**	**559,769**

Over 3 million people in the Southeast currently live in areas at or below 1 mAMSL, and hence are likely to be displaced in the next century given current projections for sea level rise (Table 3). It should be noted that the population data includes significant numbers of people in cells with an average elevation below 1 mAMSL, reflecting the nature of the sample cells, and the fact that some people do live in areas below modern sea level, in areas protected by levees such as in the lower Mississippi Delta. Even larger numbers of people live in immediately higher elevations, in the intervals from 1–2 m and 2–3 m, a pattern that differs somewhat from the archaeological and historic record, where the largest numbers of sites were found in the interval at or below 1 m in elevation. Modern populations whose occupations are not considered historic or archaeological appear to have been, on the average, occupying higher ground at greater distance from the coast. This most likely reflects infrastructure related to transportation and acquisition of potable water, although above 3 m the numbers of people, like the numbers of recorded historic and archaeological sites, also drop substantially, again reflecting a strong preference for proximity to the coast. This loss of ancient heritage will strikingly compound the injuries of climate change to indigenous peoples forced to vacate ancestral homes in coastal regions, something already happening to Native populations in the southeastern United States [111].

Appreciable terrain will also be submerged in the southeastern United States as sea levels rise, with losses in some states greater than others, with the greatest loss in Florida, which also has the longest coastal margin (Table 4). These values, of course, only tell part of the story, since the numbers of people living within these areas will be making their own decisions about how to react, individually and collectively. However slowly or rapidly sea level rise occurs, in extreme weather events storm surges and flooding will affect infrastructure, and may prompt population movement even before an area is completely submerged, with substantial impacts on cultural resources [12–17, 112–115]. As coastal terrain is flooded, increased development is likely in the regions behind the coastline, so the area of effect will extend away from the coast. What specific areas and elevations will undergo development, that is, will be occupied by displaced people and their infrastructure, will be shaped, in part, by the rate and extent of sea level rise. How this will affect local and global economies and cultures has been the subject of much recent attention, such as that given by individual southeastern states like Florida where much of the southern part of the state is at risk [116]; federal agencies like the National Park Service, which recently projected infrastructure losses for 40 coastal parks (out of a total of 117 parks in or near the coastal zone) at $40 billion [21]; and countries in the developing world, which are facing losses of potentially trillions of dollars in gross domestic product [5, 117]. Coastal zones, including large areas in the United States, many cities, and entire island nations are in immediate danger of inundation in the next century [109, 117–119]. The effects of shoreline erosion and local increases in sea level relative to land are particularly pronounced in places like coastal Louisiana [120, 121].

Sea level rise and changes in shoreline environments will not by uniformly distributed, due to variability in shoreface, beach, and substrate composition, sediment sources and sinks, freshwater sources, tidal action, and biotic communities [122–123]. Coastal landforms such as sea-islands, long a favored area for human occupation in the lower Southeast, may be especially vulnerable to both sea level rise and increased storm frequency and intensity lowering their overall height [124]. Sea level rise will also dramatically impact areas well inland, not only because that is where people will be forced to relocate, or obtain materials for dykes and similar barriers [46], but because terrestrial and marine environments, and hence human food and fresh water sources, will themselves be impacted by changes in tidal range and salinity [125–127]. The dead as well as the living will also be impacted, as sea level rise covers burial areas, a fact that appears to have shaped human worldviews in the ancient American Southeast and continues to be a subject of concern in the present [43, 67, 69]. While historic era cemeteries are not typically recorded as archaeological sites in many states unless subject to excavation, 6,897 are documented in DINAA from the 15 state regional sample, albeit very unevenly distributed due to reporting differences [73]. This is apparently a tiny subset of the estimated ca. 100,000 cemeteries present from the historic period alone in the United States [128]. Sea level rise will thus impact many burial areas ancient and modern, adding another consideration in mitigation planning [43, 45, 67, 69].

## Strategies for mitigating losses due to sea-level rise

At present, the effects of sea level rise on past cultural resources can be directly observed in the sparsity of the coastal archaeological record for the late Pleistocene and early Holocene period, during which time humans living in the Americas occupied vast areas of the continental shelf that were exposed by sea levels as much as 120 m lower than today [37–39, 61, 62]. Following a period of rapidly rising sea levels in the late Pleistocene and Early Holocene, the coastlines of the eastern United States reached near modern locations about 6000 years ago, but have still experienced fluctuations of 1 to 2 m vertically and up to several kilometers horizontally in recent millennia, with significant impacts on coastal populations [97–99, 101–102]. These regions are now directly threatened by rising waters, and the potential for the loss of thousands of years of accumulated information is significant. Given the large numbers of cultural resources threatened by sea level rise, planning possible protection and mitigation strategies should proceed with an increased sense of urgency. Many researchers and government agencies within the United States and beyond, in fact, have initiated or been developing both broad based and focused, site-specific studies on the effect of sea level rise [21–23, 25–35, 129].

One way to proceed is to use the entire known sample of cultural resources to document the numbers of properties that will be lost, by specific time period and within specific areas. Developing such a comprehensive database, of course, will be necessary, and include site records maintained by disparate state, federal, tribal, and local government agencies. This information can help to develop a triage system for cultural resources in coastal and near-coastal regions [130]. At the same time, efforts should be directed toward identifying and evaluating areas and site types currently under- or unexamined. The goal of such efforts should be to assist in the development of programs directed to the excavation, removal or relocation, and architectural documentation of critical cultural resources and resource areas. In the Southeast such efforts are appearing at the state level, including studies of significant sites or areas in Georgia [25, 131]) and Florida ([41, 66–70, 132], and collectively over large areas by federal agencies like the National Park Service [21, 28, 32]. DINAA offers a means to augment these studies by updating inventories with robust data linked to many other data sets and analytical platforms, facilitating effective resource management planning.

Data on the number of components by major temporal period located at archaeological sites within 200 km of the coast, by elevation above modern sea level, are given for the state of South Carolina in Table 5. The numbers of sites in each elevation interval correspond to the state totals, since they are derived from the same site file data set in DINAA (Table 1), but the numbers of components are invariably higher, in some cases much higher, because some sites were repeatedly visited and are multicomponent. In some cases individual occupations can be quite specifically identified to temporal period while others can be only generally identified to age, perhaps no more specifically than to a categorization as precontact or historic. It should be noted that comparable tables can be generated for each state in the region; South Carolina was chosen as an example for illustrative purposes, and to show the potential of DINAA.

**Table 5:** Archaeological site and component loss in South Carolina due to sea level rise within 200 km of the coast. **Data: [73].** PI = Paleoindian. EA = Early Archaic, MA = Middle Archaic, LA = Late Archaic, AA = Any Archaic, EW = Early Woodland, MW = Middle Woodland, LW = Late Woodland, AW = Any Woodland, M = Mississippian, LP = Late Prehistoric, UP = Unknown Prehistoric, CEP = Contact Era/Protohistoric, 16^th^ = 16^th^ Century Historic, 17^th^ = 17^th^ Century Historic, 18^th^ = 18^th^ Century Historic, 19^th^ = 19^th^ Century Historic, 20^th^ = 20^th^ Century Historic, UH = Unidentified Historic.

**mAMSL**	**PI**	**EA**	**MA**	**LA**	**AA**	**EW**	**MW**	**LW**	**AW**	**M**	****
Below 0	4	14	8	45	0	56	77	50	0	26	
0 to 1 m	3	11	9	122	0	221	329	209	1	126	
>1 to 2 m	0	4	3	57	0	108	170	97	0	53	
>2 to 3 m	2	8	5	130	0	205	296	165	5	94	
>3 to 4 m	2	3	2	54	0	75	133	53	1	35	
>4 to 5m	0	7	6	63	0	98	150	70	3	36	
>5 to 10 m	3	42	60	365	6	601	975	508	13	279	
>10 to 15 m	4	32	32	155	6	171	271	129	17	66	
>15 to 20 m	5	31	35	76	3	87	126	67	6	26	
>20 to 25 m	10	40	56	78	3	100	117	51	4	32	
>25 to 30 m	10	50	50	73	0	86	117	63	0	18	
>30 to 50 m	28	136	141	193	3	180	197	140	3	67	
>50 m	24	202	276	337	0	411	523	206	0	123	
**Sample Total**	**95**	**580**	**683**	**1,748**	**21**	**2,399**	**3,481**	**1,808**	**53**	**981**	
										**Component**	**Site**
**mAMSL**	**LP**	**UP**	**CEP**	**16th**	**17th**	**18th**	**19th**	**20th**	**UH**	**Totals**	**Totals**
Below 0	0	130	0	2	8	98	219	108	108	953	547
0 to 1 m	0	426	0	6	38	275	574	293	179	2,822	1,551
>1 to 2 m	0	166	0	0	15	130	252	128	93	1,276	619
>2 to 3 m	0	313	0	3	24	235	526	280	158	2,449	1,350
>3 to 4 m	0	86	0	0	7	77	153	76	42	799	425
>4 to 5m	0	117	0	2	10	74	154	85	54	929	471
>5 to 10 m	0	658	0	0	34	483	893	658	317	5,895	3,090
>10 to 15 m	0	172	0	1	2	99	200	185	83	1,625	909
>15 to 20 m	0	99	0	0	2	35	148	148	67	961	537
>20 to 25 m	0	205	0	0	2	37	212	220	65	1,232	656
>25 to 30 m	0	170	0	0	0	18	161	215	48	1,079	646
>30 to 50 m	0	491	0	1	1	70	433	489	125	2,698	1,678
>50 m	0	977	0	2	0	84	806	1,008	156	5,135	3,681
**Sample Total**	**0**	**4,010**	**0**	**17**	**143**	**1,715**	**4,731**	**3,893**	**1,495**	**27,853**	**16,160**

Land use patterns are highlighted when examining individual state data by time period, and help to determine if survey coverage or research biases have affected the estimated number of existing sites in coastal areas. Few early prehistoric Paleoindian through Middle Archaic period components, for example, are found in coastal (i.e., low elevation) areas in South Carolina, compared to the much larger number of later precontact and historic components. With greatly lowered sea levels during these earlier periods, the coast would have been much farther away, perhaps making these areas less attractive for settlement. Likewise, given the intense occupation in coastal areas after sea levels largely stabilized in the region during the later Mid-Holocene [133–136], it is not surprising that large numbers of components are found in close proximity to modern sea level. Even in this area, appreciable variability in location exists, due to the effects of ca. 1–2 m fluctuations in sea level in recent millennia ([97–99, 101, 102]. Interestingly, within the South Carolina sample the larger regional pattern holds, in that that large number of components are found within 1 m of modern sea level, and far fewer above 3 m in elevation, reinforcing the conclusion that people over the last several thousand years lived in close proximity to the coast, albeit shifting location as needed to accommodate the fluctuations in sea level of plus or minus 2 m or so that have occurred.

Resource managers will need to evaluate sites in large numbers to determine which ones to preserve, protect, or mitigate. This is no different than what modern cultural resources management deals with on a regular basis, only here we call for consideration of the entirety of the coastal record as one data set, rather than on an individual case-by-case basis. Effective systems of management, including triage and mitigation, can only be developed when we have an accurate understanding of the cultural resources in an area, and where critical gaps in that knowledge exist. Existing databases need to be completed or developed and subsequently linked to systems like DINAA, while strict protections for sensitive location and other information are maintained. Many cultural resource databases reflect incomplete coverage of a geographic area or contain only particular kinds of data. A recent exemplary study of the effects of sea level rise on National Park Service coastal parks, for example, excluded most known archaeological resources because they were not part of the Facilities Management Software System database listing assets requiring routine maintenance within each NSP unit [21]. Improving and linking dispersed databases, and rendering them interoperable for research and management purposes, will allow management decisions to proceed with much larger and more representative samples.

Archaeologists and land managers need to be aware that cultural resources face specific threats, and that sea level rise will impact resources differently in different areas, depending on geomorphological factors like shoreline shape and slope, the underlying matrix, the nature of the archeological deposits, and a range of other variable associated with the cultural properties [22–26, 28–30, 123, 137]. For example, some shell middens dating to the Mid-Holocene have already witnessed episodes of submergence and exposure, but remain at partially intact in coastal marshlands of the Southeast (e.g., [97, 98, 136], suggesting sea level rise does not necessary always equate with the total destruction of all types of resources. The circumstances favoring preservation or loss of coastal sites will need to be carefully evaluated on an individual or class basis [22, 130]. Resources directed to cultural resources will undoubtedly change as environmental conditions change, and historic preservation specialists will continue to have a major role in preserving our cultural heritage [26, 114–115, 129, 138]. Guidance for resource managers on how to deal with the impacts of climate change is clearly needed, and action directed to these ends is underway in federal agencies like the US National Park Service [21, 28, 29, 139, 140] as well as international governing bodies like the United Nations [141, 142].

Effort should be directed to making sure our inventories of cultural resources are accurate, adequate, as complete as possible, and linked together with interoperable data elements, so the information can be utilized to prioritize preservation projects and research problems by site type and risk level, allowing the most pressing needs in resource preservation to be addressed effectively. More sites should be evaluated for placement on the NRHP; at present, in some circumstances only formal listing offers any hope of preservation or mitigation. Resources should be directed to evaluating sites in large numbers, as has happened with southeastern coastal shell ring and midden sites [132, 135, 136]. The economic costs of mitigating cultural resource loss through excavation, relocation, or architectural documentation should be considered thoroughly and incurred conscientiously, as it is well known that public funding for historic preservation efforts is often difficult to acquire, limited in quantity, and requires a high level of justification.

Ultimately, what will be needed is a commitment, like that last seen in the Great Depression, to document that which will be lost if the effects of sea level rise are not mitigated. This time, instead of rescuing information from sites in reservoir floodpools as was done by the Tennessee Valley Authority [143], or deliberate economic recovery or tourist-industry focused make-work projects like those in the Macon, Georgia area [144], much of the work will need to occur in coastal areas or where the resettlement of displaced populations will occur. The Cape Hatteras lighthouse relocation was expensive and technically challenging, but offers an excellent example of what can be done when resources are made available [51]). Consideration may have to be given to relocating or constructing protective barriers for other such monuments, like the Castillo de San Marcos and Ft. Matanzas in St. Augustine, for example, or the Lincoln and Jefferson Memorials [21, 28]. The solution to addressing the effect of sea level rise on major centers of heritage, like the nations’ capitol, Boston, New York, or Washington, to list just a few of the threatened cities that will receive consideration ([119]), would probably be the construction of sea walls and similar projects, whose cost is projected to be far less than the damage caused by flooding [46]. While these kinds of projects would cause massive damage to cultural resources in the construction zone, including where fill/retaining wall materials came from, their loss could be better accepted given an effective assessment of the totality of the resources affected by sea level rise. Sites in heavily developed, low lying areas may in fact be at less risk, because there will be added effort taken to protect those areas. An NRHP eligible site or structure in central New Orleans is probably more likely to be protected by new sea walls or levees than a shell ring on a low, relatively undeveloped southeastern coastline.

This analysis assumes sea level rise will destroy cultural resources. Of course, depending on the rate and rapidity of rise, it may only submerge these resources, with the extent of damage or loss uncertain. Some studies have shown that sea level fluctuations may not totally destroy cultural resources; much depends on the rapidity and frequency with which submergence or exposure may occur (e.g., [37, 38, 127, 136]. More such studies are critically needed, since preservation in place may be our only option for most sites, unfortunately by default. What will be preserved is important to determine, because it will mean resources can be directed to other, more vulnerable site types. Some of these sites may be accessible using underwater archaeological methods in the future, meaning mitigation should be directed to site types unlikely to survive sea level rise or storm surges. Finally, we need to be thinking not just about sites and architecture, but also about the long term curation of physical collections and records. Storing the archival records and collections within one or even several meters vertical elevation above modern sea level will need to be rethought, since such actions can no longer be considered a viable means of ensuring effective curation in perpetuity.

## Conclusions

Although the scientific community recognizes the profound impact of humans on the natural environment in recent centuries, few institutions fund the investigation of long-term human-environmental interactions through database development like DINAA. The initial data collection and integration phase of DINAA has been undertaken largely voluntarily by project team members at several institutions, together with limited funding from the Archaeology Program of the National Science Foundation. This has allowed us to develop a proof-of-concept framework integrating archaeological data from 15 states [72, 73], for linkage to environmental and collections data sets. DINAA demonstrates how a truly continental archaeological database useful for research, resource management, and public education can be developed, and how it can be maintained and updated on a regular schedule by a sustainable community of scholars and stakeholders.

Linking archaeological site files and other data sets at broad scales catalyzes research across disciplines, promoting more holistic understanding of both human adaptation and environmental impacts. As multidisciplinary databases addressing sea level and other forms of global change are developed, the role of cultural resources are increasingly coming to be regarded as a critical factor when planning mitigation strategies [19, 27, 148, 149]. DINAA, through its adoption of an open data policy (within limitations regarding sensitive information), promotes information sharing and integration, not only of archaeological but paleoenvironmental, biogeographical, physiographic, and other data characterizing our environment. Within archaeology such approaches to data management are increasingly viewed as not only good science, but an ethical obligation [150]. DINAA has open-ended applications allowing researchers, land managers, and interested members of the public to examine the nature and scale of human responses to the dramatic fluctuations in temperature, biota, and sea level that have occurred over the ca. 15,000+ years people have lived in the Americas, and help inform our understanding of possible human responses to similar changes predicted for the future, questions of critical importance.

Hopefully there will be time to implement these suggestions. However, changes in sea level may be far greater and occur far faster than currently predicted. Delay in thinking about these matters and in seeking solutions accomplishes nothing. Developing data infrastructure like DINAA is crucial to multidisciplinary analyses linking differing kinds and sources of data together and rendering them interoperable. By facilitating the mapping of archaeological sites over time and at varying geographic scales, showing where people were on the landscape and how they reacted to changes in climate and biota, tools like DINAA are useful to addressing research and management concerns. These include helping people gain a much greater appreciation for American history and culture, and protecting the vulnerable heritage of indigenous communities. Linked data can be used to explore the impact of sea level rise on cultural and historical resources. The effects of sea level rise on cultural resources is intimately linked to the humanitarian and economic issues that need to be faced in all crises [44, 145]. Cultural resources, promoting an awareness of and appreciation for our heritage, are essential to our well-being, and a continuing source inspiration [26, 146, 147]. Population relocation and new infrastructure required to cope with sea level rise, we have seen, will have severe negative impacts on coastal and near-coastal cultural resources. Given the investment humanity has made in these areas, efforts should be directed to preventing and, if this is not possible, managing potential losses.

Cyberinfrastructure development is a critical part of 21^st^ century archaeology, and projects like DINAA will make archaeological data increasingly useful and relevant to research, management, and public educational efforts. Data-driven archaeology can provide unparalleled insights into long-term human-environmental interactions, enabling archaeology to more fully participate in the efforts directed to understanding the impacts of climate change. Such knowledge is critical to making well informed forecasts and policy decisions about the consequences of rapid climate change, extreme weather events, and burgeoning populations, factors that will shape our civilization profoundly in the coming decades. While legal and ethical restrictions require that we safe-guard the precise location data behind this study (which is available from the agencies maintaining it [151]), DINAA makes data openly available with a lower level of spatial resolution to enable at least partial replication of these analyses, and most critically, to enable researchers in many fields of study to try other applications, using a framework built on information from the past to project trends forward in time.

Our species has witnessed comparable periods of dramatic climate change in the past, and understanding how we responded can provide valuable lessons, and hope, for the future. Indeed, these are some of the greatest lessens archaeology can teach us, by providing information about how past human response, and resilience, as we move forward into an increasingly uncertain world.
